# Silica nanolayer coated capillary by hydrothermal sol–gel process for amines separation and detection of tyramine in food products

**DOI:** 10.1038/s41598-022-11078-y

**Published:** 2022-05-06

**Authors:** Apinya Obma, Pattamaporn Hemwech, Sittisak Phoolpho, Rawiwan Bumrungpuech, Supa Wirasate, Sulawan Kaowphong, Prapin Wilairat, Rattikan Chantiwas

**Affiliations:** 1 Department of Chemistry, Faculty of Science, Mahidol University, Rama VI Rd., Bangkok, 10400 Thailand; 2 Center of Excellence for Innovation in Chemistry and Flow Innovation-Research for Science and Technology Laboratories (FIRST Labs), Faculty of Science, Mahidol University, Rama VI Rd., Bangkok, 10400 Thailand; 3 Center for Surface Science and Engineering and Rubber Technology Research Center, Faculty of Science, Mahidol University, Salaya, Nakhon Pathom, 73170 Thailand; 4 Department of Chemistry and Environmental Science Research Center (ESRC), Faculty of Science, Chiang Mai University, Chiang Mai, 50200 Thailand; 5 Analytical Sciences and National Doping Test Institute, Mahidol University, Rama VI Rd., Bangkok, 10400 Thailand

**Keywords:** Analytical chemistry, Techniques and instrumentation

## Abstract

A hydrothermal sol–gel method for reproducible formation of silica nanolayer on the wall of silica capillaries was developed for electrochromatography. The formulation was optimized by observation of uniform gel formation on an optical microscope. The variables of the formulation include types of solvent, water-TEOS ratio, CTAB and urea contents, and mixing method. The procedure produced a coating of silica *ca.* 100 nm thick layer on the wall of the capillary. Surface morphology of the coating was characterized by SEM, contact angle and chemical composition by FT-IR spectroscopy and X-ray powder diffraction. The coating reduced the electroosmotic mobility producing enhanced separation performance. Eight standard amines (including tyramine and benzhydrylamine, as an internal standard) were separated with peak resolution *R*_s_ ≥ 2 for all adjacent peaks and plate number *N* ≥ 3.0 × 10^4^ m^-1^. Calibration was linear from 5 to 200 µg L^-1^, with r^2^ > 0.9985 and instrumental LOD of 4.9 μg L^-1^. Five samples of food products were diluted and analyzed for the amines using the coated capillary and only tyramine was detected. Intra-day and inter-day precisions were less than 1.2%RSD. Percent recoveries of spiked tyramine in samples were 95 ± 3 to 106 ± 7% (*n* = 3).

## Introduction

Development towards highly efficient separation has been a focus of research in capillary electrophoresis and chromatography^1,2^. For the former, studies of electrolytes and additives for modifying the electroosmotic flow (EOF) have been reported^3^. Another approach is to coat the inner wall of the silica capillary with covalently bonded chemical compounds or with a nanometer layer of material^4–6^. The latter can be carried out by polymerization or sol–gel formation. Review articles of these methods have been published^7,8^.

The coatings on the inner wall of capillaries comprise a variety of materials, such as metal- framework^9^, zinc oxide nanoparticles^9^ and nanocellulose crystals ^10^. Methods of coating include layer-by-layer assembly^11^, polymerization^12^ and sol- gel process ^13^. If the resulting coating is porous, the capillary is often named as a porous layer open-tubular (PLOT) capillary. Sol–gel processes are commonly employed for the formation of porous layer on the inner wall of a silica capillary. It is simple, convenient and efficient, involving only hydrolysis and polycondensation of an alkoxide precursor. Removal of the solvent gives the xerogel or aerogel and after a heating step the final solid structure^14^. Table 1 lists the compositions of the various sol–gel mixtures and conditions for gel formation, together with the characterization of the coating and application of the coated capillary^10,13,15–17^.

**Table 1:** Previous reports of the various sol–gel mixtures and conditions for gel formation, together with the characterization of the coating and application of the coated capillary.

Ref	Coating conditions		Sol mixture preparation		Characterization	Separation
	Equipment and conditions	Thermal treatment (temp., time)	Mixing tool	Temp., time		
^15^	Nitrogen pressure at 689.4 kPa (20 min)^a^	170 °C, 1 h	Vortex	RT, 5 min	NA	-Basic proteins and nucleotides
^16^	Aspirator vacuum system by dipping one end in solution^b^	50 °C, 24 h	Ultrasonic bath	RT, 30 min	SEM	-Nitrate, nitrite, aromatic acids, basic drugs and proteins
^10^	Peristaltic pump at 1.0 mL min^-1^ (5 min)^c^	RT, 5 min	Magnetic stirrer	45 °C, 2 h	SEM XPS	Enantiomers (phenylalanine, tryptophan and tyrosine)
^17^	Dip coating cycle multiple times with controlled pulling speed 4 mm/sec (up to 6 cycle)^d^ Note: Equipment not mentioned	RT, 10 min	Magnetic stirrer	0 °C, 4 h	SEM FT-IR	Enantiomers (phenylalanine, tyrosine, tryptophan, phenethyl alcohol, 1-phenyl-2-propanol, and Troger’s base)
^13^	NA^e^	70 °C, 16 h	Magnetic stirrer	RT, 15 min	SEM XPS FT-IR	beta-blockers, polycyclic aromatic hydrocarbons and peptides
This work	Syringe pump at flow rate of 3.0 µL min^-1^ (5 min)^f^	70 °C, 4 h	Ultrasonicator (2 kHz, 20 watts)	RT, 30 s	FT-IR XRD *In-situ* SEM CA	Separation of eight amines (histamine, benzylamine, phenylethylamine, tyramine, benzhydrylamine, dopamine, propranolol and atenolol)

*RT* Room temperature, *NA* Not available, *SEM* Scanning electron microscope, *XPS* X-ray photoelectron spectroscopy, *FT-IR* Fourier-transform infrared spectroscopy, *XRD* Powder X-ray diffraction, *CA* Contact angle measurement.

^a^Sol-gel formulation: 400 µL TEOS, 500 µl methyl formate, 100 µL formamide, 20 mg Ucon 75-H-90000, 30 µL hexamethyldisilizane, 20 µL 0.1 M dicumyl peroxide in pentane and 100 mL of 0.5 M ethanolamine solution in methanol–deionized water (6:1 v/v).

^b^Sol-gel formulation: 500 µL TMOS,180 µL water, 15 µL 0.1 M HCl, 340 µL TMSPTMA.

^c^Sol-gel formulation: 0.05 g DMPC/NCCs-silane, 13 mL pyridine, 1.5 mL TEOS and 1.0 mL absolute ethyl alcohol mixture solution, 0.05 g CTAB and 0.125 mL HCl and 0.18 mLHF.

^d^Sol-gel formulation: 0.1272 g nano-ABDMPC bearing 3-(triethoxysilyl)propyl residues in 10 mL pyridine, 1.2 mL TEOS, 1.0 mL absolute ethyl alcohol mixture solution, 0.04 g CTAB, 0.2 mL 12 M HCl and 0.32 mL 0.2 M HF.

^e^Sol-gel formulation: 500 µL TEOS, 3000 µL cyclohexane, 3000 µL water, 100 mg CTAB, 60 mg urea and 92 µL 1-pentanol.

^f^Sol-gel formulation: 500 µL TEOS, 3000 µL cyclohexane, 3000 µL water, CTAB 100 mg, 60 mg urea, 440 µL 0.1 mM acetic acid and 92 µL 1-pentanol.

However, the method of using the sol–gel process to form a consistent thin layer on the inner wall of a 50 µm i.d. capillary is still a subject of study ^13^. Thus, the objective of this study is to develop a sol–gel formulation and a simple process to give coatings with reproducible nanometer thickness of the layer. The coated capillary will provide stable and more efficient performance for capillary electrochromatography (CEC) application.

Tyramine is a biogenic amine commonly found in food and beverages^18,19^. However, its accumulation is of health concern. High amount of tyramine, e.g. 200–2000 mg per meal, can produce high blood pressure or trigger migraine attack. Consumption of low levels of tyramine (6 mg per meal) has been reported to produce side effects and high blood pressure in patients taking monoamine oxidase inhibitors as antidepressant^19,20^ and avoiding tyramine in food and drink products are necessary for such patients^21,22^. Methods for the determination of tyramine in beverages and food have been reported using high performance liquid chromatography with UV detection (HPLC–UV)^23,24^. These methods involve solid-phase extraction^23^ and derivatization^25^. Determination of tyramine employing HPLC and GC methods have limits of detection of 0.38 μg L^-1^^26^ and 1 μg L^-1^^27^, respectively. Capillary electrophoresis with UV absorbance or fluorescence emission has been used, with the latter requiring derivatization procedure ^28^. Although UV absorbance has limitation of sensitivity due to the very short optical path length, the CE method^23,29,30^ has sufficient sensitivity for analysis of tyramine at the mg L^-1^ level (see Table 2). However, detection at lower levels will be required for the determination of tyramine in food and beverages consumed by patients taking monoamine oxidase inhibitor antidepressant drugs^20^.

**Table 2:** Previous methods for tyramine analysis using CE-UV detection with number and types of amine, the analytical performance (linear range, %RSD of migration time, LOD and LOQ and recovery), internal standard employed, separation time and application of sample analysis.

Parameters	^23^	^29^	^30^	This work
Number of amines (amine analytes)	Two amine compounds (histamine, tyramine)	Three amine compounds (histamine, 2-phenylethylamine, tyramine)	Eight amine compounds (trimethylamine, putrescine, cadaverine, spermine, tryptamine, spermidine, phenylethylamine, tyramine)	Eight amine compounds (histamine, benzylamine, phenylethylamine, tyramine, benzhydrylamine, dopamine, propranolol and atenolol)
**Analytical performance**				
Linear range	5–1000 mg L^-1^	0.2–10 mg L^-1^	2–50 mg L^-1^	5–200 μg L^-1^
%RSD of migration time	NA	0.80 (*n* = 5)	NA	< 1.8 (*n* = 5)
LOD and LOQ	6 mg L^-1a^ and NA	0.37 mg L^-1b^ and 0.55 mg L^-1b^	0.5 mg L^-1c^ and 1.5 mg L^-1c^	4.9 μg L^-1d^ and NA
Recovery	96–102	96	NA	95–106
Internal standard employed	NA	NA	NA	Benzhydrylamine
Separation time	~ 9 min	~ 30 min	~ 15 min	~ 7 min
Application of sample analysis	Fish, cheese, meat products and vegetarian product	Wine	Pork, chicken, beef, salmon, hake, ham and sausages	Food and drink products; beer, wine, balsamic vinegar and hard cheese^e^

*NA* Not available.

^a^Calculation of LOD and LOQ not shown.

^b^LOD and LOQ were calculated by QC Expert 2.5 (IUPAC method).

^c^LOD and LOQ were calculated from 3 × SD and 10 × SD of the blank signal.

^d^LOD was calculated from 3 × (SD of intercept)/slope of calibration line.

^e^Extraction before analysis (Supplementary Information E).

In this work, the coated capillaries are tested for efficiency of separation of eight amine compounds including tyramine and benzhydrylamine (used as the internal standard). The capillaries are then applied for the detection of tyramine in various food products using dilution as the only sample preparation step. The sensitivity of the analysis and advantages of the method are compared with previous works.

## Experimental

### Chemicals

#### Sol–gel experiments

Tetraethyl orthosilicate (TEOS; 99% assay, GC grade), 1-pentanol (99% assay, GC grade) and 1-octanol (96% assay, GC grade) were supplied from Sigma Aldrich (St. Louis, MO, USA). Cetyltrimethylammonium bromide (CTAB; 98% assay, molecular biology grade) and sodium hydroxide were obtained from Merck (Darmstadt, Germany). Urea (99% assay, AR grade) was purchased from Kemaus (Cherrybrook, NSW, Australia). Glacial acetic acid (99.8% assay, AR grade) and cyclohexane (99.7% assay, AR grade) were from QREC™ (SV Medico, Hat Yai, Songkhla, Thailand). Benzene (99.8% assay, AR grade) was supplied by Panreac Quimica SLU (Barcelona, Spain). Methanol (99% assay, AR grade), ethanol (AR grade), pentane (99% assay, AR grade), hexane (HPLC grade), *tert*-butyl methyl ether (TBME, HPLC grade), dimethyl sulfoxide (DMSO; AR grade) and toluene (99.5% assay, AR grade) were from RCI Labscan (Bangkok, Thailand). Ultrapure water (18.0 MΩ.cm) was produced from Milli-Q Advantage A10 water purifying system (Merck, Darmstadt, Germany).

#### CE experiments

*L*-Histamine (B grade) was obtained from Calbio-chem (San Diego, CA, USA). Benzylamine (98% assay, GC grade), phenethylamine (99% assay), tyramine (98% assay), dopamine hydrochloride (98% assay) and benzhydrylamine (97% assay) were purchased from Sigma-Aldrich (St Louis, MO, USA). Atenolol and propranolol (Reference Standard) were supplied from Bureau of Drug and Narcotics (Department of Medical Sciences, Nonthaburi, Thailand). Phosphate running buffer was prepared from orthophosphoric acid (85% assay, UNIVAR® Analytical Reagents (Ajax Finechem Pty Ltd, Australia).

### Food and drink products

A variety of food and drinks samples were selected. They include beer, wine, balsamic vinegar and cheese. The products were bought from a supermarket in Bangkok, Thailand. Liquid samples were diluted with ultrapure water before analysis. The cheese sample was prepared by solvent extraction method^31^. Details of this procedure are given in Supplementary Information E.

### Formation of silica layer coating on capillary wall by hydrothermal sol–gel process

**Figure 1:**
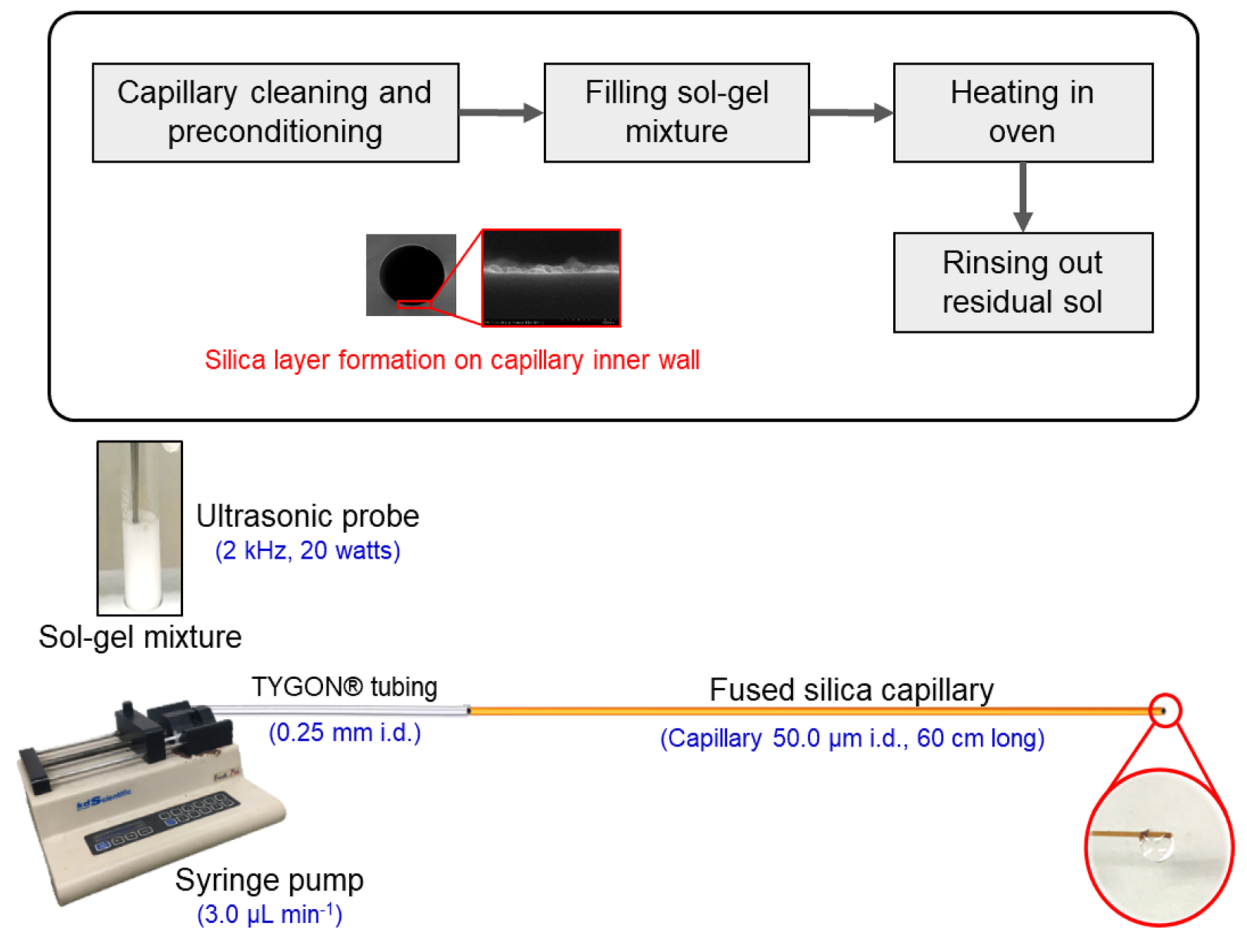
Schematics of cleaning, filling with sonicated sol–gel mixture, heating and final rinsing of capillary.

Figure 1 is the schematics of the procedure for the hydrothermal sol–gel coating of the inner wall of a capillary. A fused silica capillary (360 µm o.d. and 50 µm i.d.) is first cleaned and preconditioned by flushing with MeOH-H_2_O (50% v/v) for 5 min, then with 1.0 M NaOH for 30 min and finally with ultrapure water for 5 min. A syringe pump (model CEC-MSP-001, Unimicro Technologies, CA, USA) with a 0.5-mL syringe connected to 0.25-mm i.d. TYGON® tubing is used with the flow rate set at 3.0 µL min^-1^. The conditioned capillary is then dried with a nitrogen stream at room temperature for 15 min. The sol–gel mixture is then prepared using the optimized formulation, viz*.* cyclohexane (3.00 mL), TEOS (500 µL), ultrapure water (3.00 mL), CTAB (100 mg), urea (60.0 mg), 0.10 mM acetic acid (440 µL), and 1*-*pentanol (92 µL). The mixture is homogenized using the ultrasonic probe (2 kHz, 20 watts) for 30 s. The mixture is then immediately pumped into the capillary at the flow rate of 3.0 µL min^-1^ for 5 min using the syringe pump. The ends of the capillary are then sealed with GC septa and the capillary heated in a gas chromatography oven (HP 6890A, Agilent, Santa Clara, CA, USA) for 4 h at 70 °C (see Fig. S1 in Supplementary Information A for the temperature program). It is found that heating for longer than 4 h resulted in the gel clogging the capillary. After cooling, the capillary is flushed with ethanol followed by water for 5 min each, to remove the residual reagents, and dried with a nitrogen stream for 15 min. The silica nanolayer coated capillaries are stored at 50 °C in a glassware drying oven until employed.

### Procedure for selection of sol–gel formulation for capillary coating

Experiments were first carried out to find a suitable formulation that will give uniform formation of silica nanolayer on the wall of silica capillary. The various formulations and mixing methods are given in Table S1 in Supplementary Information A. Each sol–gel mixture (ca. 7 mL) is homogenized either by vortex mixing (VTX-3000L model, LMS Ltd., Tokyo, Japan) or by ultrasonication (TT13 probe, HD2200 model, BANDELIN, Berlin, Germany).

**Figure 2:**
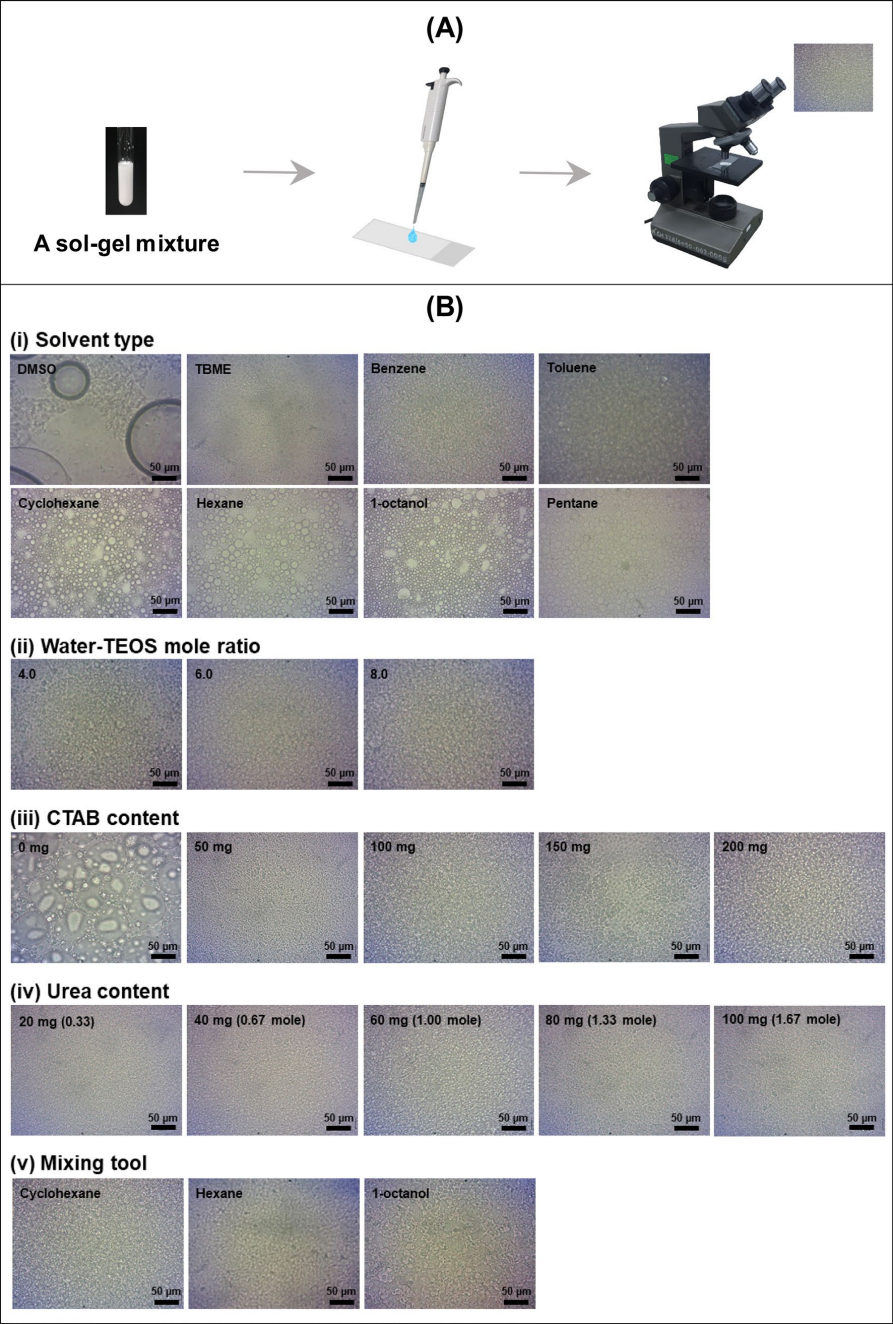
(**A**) Schematic of method for preparing sol–gel sample for observation on optical microscope. (**B**) Images recorded from an optical microscope showing features of gel for various sol–gel formulations. The parameters are (i) solvent type, (ii) water-TEOS mole ratio, (iii) CTAB content, (iv) urea content and (v) method of mixing.

For each formulation, a small amount of the mixture is deposited on a microscope glass slide and covered with a cover slip. The inspection of sol–gel formation is followed with an optical microscope (CH-Series, Olympus America Inc., PA, USA). Images are recorded at 10 × magnification and analyzed using DinoCapture 2.0 software (AnMo Electronics Corporation, New Taipei, Taiwan). The criteria for selection of the suitable sol–gel formulation and mixing method are the size of the immiscible droplets, their distribution and the time for the formation of the droplets (Fig. 2A). The remaining mixture is visually monitored for gel formation with intermittent shaking of the test tube. The gelation time is taken as the period from the completion of the mixing step to the visual observation of gel formation.

### Characterizations of sol–gel coating material

Characterizations were carried out using Fourier transform infrared spectrometer (FT-IR), X-ray powder diffractometer (XRD), scanning electron microscope (SEM) and contact angle (CA) measurements. The coating material was prepared from the sol–gel composition mixture as described in “Formation of silica layer coating on capillary wall by hydrothermal sol-gel process” section.

**FT-IR**: The IR spectra of the sample powder was measured using attenuated total reflection (ATR) accessory on a Bruker instrument (INVENIO®) in the range of 4000–400 cm^-1^.

**XRD**: The structure of the silica coating material was determined by powder X-ray diffraction using Bruker X-ray diffractometer (D2 PHASER) with Cu Kα beam (λ = 1.54184 Å) for angle 2*θ* from 5.0 to 70.0°.

**SEM**: Surface morphology of the coated layer at the silica wall was observed *in-situ* by field emission scanning electron microscope (FE-SEM, JSM-6335F model, JEOL Ltd., Tokyo, Japan) operated at accelerating voltages of 5.0 and 15.0 kV. The coated capillaries were cut horizontally and at a bevel angle. Details of the procedure are given in Fig. S2 in Supplementary Information B. Prior to SEM imaging, the nanolayer coating was deposited with a thin film (~ 10 nm) of conductive gold layer to prevent charging of the surface layer before mounting for the measurements.

**Contact angle** (**CA)**: A sessile water drop contact angle measurements were performed using ultrapure water and a contact angle goniometer (model G-1, KRÜSS GmbH, Hamburg, Germany) equipped with a CCD camera (iPhone 6s). A 5.0 µL volume of water was placed on the substrate surface (either non-coated or coated glass slide) using a micro syringe pipette. Images of the water drop were taken immediately after depositing on the glass slide surface. The left and right contact angles of the water drops were measured (three replicates, *n* = 6) using ImageJ software with Plugin (see Supplementary Information B for the operation steps of the measurements).

### Capillary electrophoresis with UV detection

The capillary electrophoresis instrument was assembled in-house, comprising a high voltage power supply (Spellman CZE1000R, Hauppauge, NY, USA) and a micro-cell UV detector (785A UV detector, Applied Biosystem, CA, USA). Fused silica capillaries (360 µm o.d. and 50 µm i.d.) were from Polymicro Technologies (Phoenix, AZ, USA). A length of 60.0 cm, with effective length of 38.0 cm, was used. Conditioning of non-coated and coated capillaries were carried out by sequentially flushing with 0.1 M NaOH, ultrapure water and running buffer for 5 min each, at flow rate of 3.0 µL min^-1^, using the syringe pump. The separation conditions are electrokinetic injection for 3.0 s at 400 V cm^-1^, applied potential of 400 V cm^-1^ and UV detection at 200 nm. Phosphate running buffer (20.0 mM, pH 2.5) was prepared daily by diluting stock 500.0 mM phosphoric acid and adjusting to pH 2.5 with 1.0 M NaOH. Stock standard solutions of the eight amines (histamine, benzylamine, phenethylamine, tyramine, dopamine, propranolol, atenolol and benzhydrylamine (IS)) were prepared at 1000 mg L^-1^ each. All solutions were stored at 4 °C. Working standard solutions were freshly prepared by dilution with ultrapure water.

## Results and discussion

### Selection of formulation of sol–gel mixture for hydrothermal sol–gel formation

Various formulations of sol–gel mixture were investigated for reproducibility and uniformity of a coated layer. Formulations for sol–gel formation can be composed of various components such as silicon alkoxide precursor (*e.g.* tetraethyl orthosilicate (TEOS), tetramethyl orthosilicate (TMOS), tetrapropyl orthosilicate (TPOS)^32^, organic solvent (e.g. alcohol, ether), gelating agent (e.g. urea, urea derivative)^33^, stabilizer (e.g. cationic surfactant)^34^, acid or base catalyst^32^. The mole ratio of water to silicon alkoxide is also an important factor^32^.

#### Optical microscopy evaluation of sol–gel mixture

A sol–gel formulation selected from previous reports was used as the starting formulation^13^. This comprises 500 µL TEOS (0.0259 mol), 3 mL water (0.1667 mol, water-TEOS mole ratio, 6:1), 3 mL organic solvent, 100 mg CTAB, 60 mg urea, 440 µL 0.10 mM acetic acid, and 92 µL 1*-*pentanol (total volume of *ca.*7 mL). The mixture was either vortexed (3000 rpm, 1 min) or sonicated with a probe (2 kHz, 20 watts) for 30 s. A small amount was inspected under an optical microscope (see “Procedure for selection of sol-gel formulation for capillary coating” section and Fig. 2A) and the gelation time of the remaining mixture recorded (see “Procedure for selection of sol-gel formulation for capillary coating” section).

The amount of the various components and the method of mixing (vortex or sonication), were varied in a univariate manner to find the optimal formulation as observed by optical spectroscopy (see “Procedure for selection of sol-gel formulation for capillary coating” section). Observation of sol–gel coating by optical microscopy was previously employed to study uniformity of sol–gel deposition on silicon substrate^35,36^. Summary of the results are described below.

##### (i) Solvent type

A 3.0 mL aliquot of eight solvents were tested, viz*.* DMSO, TBME, benzene, toluene, cyclohexane, hexane, 1-octanol, and pentane, with vortex mixing for 30 s. The optical images for the various solvents are shown in Fig. 2B(i). Formation of uniform droplets was observed only for cyclohexane, hexane, 1-octanol and pentane, respectively. The time for formation of gel in the bulk mixture ranged from less than one minute (for pentane) to more than 30 min. However, samples of the sol–gel mixture containing cyclohexane gave more stable immiscible droplets than for 1-octanol or hexane. Thus 3.0 mL of cyclohexane was used in the following study.

##### (ii) Water-TEOS mole ratio

The mole ratio of water to TEOS has a significant effect on the rate of the phase change from sol to gel transition and the gelation time^32^. Formulations with different mole ratios (4.0, 6.0 and 8.0) were prepared by fixing the amount of TEOS at 0.0259 mol (500 µL) and varying the amounts of water (2, 3 and 4 mL). The organic solvent was cyclohexane (3 mL) and ultrasonication was employed. Optical images for the three mixtures of the mole ratios are shown in (Fig. 2Bii). Uniform distribution of small immiscible droplets are observed. The gelation times are 5, 5 and 1 min, respectively. The water-TEOS mole ratio of 6.0 was selected for the next study as it provided time (5 min) for pumping into the capillary before the gel formation is complete.

##### (iii) CTAB content

The amount of CTAB (a stabilizing agent) affects the pore size and also the rate of gel formation^34^. The amount of CTAB was varied from 0 to 200 mg in steps of 50 mg. Sonication was employed in the mixing step. The optical images in Fig. 2B(iii) show that without CTAB the droplets are non-uniform in size, with large droplets formed. With increased amount of CTAB there is more uniform distribution of droplets, with the droplet size decreasing with increasing amount of CTAB (see Fig. 2Biii). Moreover, there was a steady decrease in the gelation time from 30 to 1 min. A 100 mg amount of CTAB, with gelation time of 5 min, was selected for the following study.

##### (iv) Urea content

Urea is a porogenic agent, affecting both the pore size and gelation time^33^. The amount of urea was varied from 20 to100 mg in increment of 20 mg. The optical images of the gel mixtures in Fig. 2B(iv) show little changes in the size and distribution of the droplets with the amount of urea. Gelation time was less than 5 min for urea content greater than 80 mg. Therefor, 60 mg urea was selected for the optimal coating formulation.

##### (v) Mixing procedure

The technique used for mixing the formulation has an effect on the dispersion of the microdroplets in the sol–gel mixture. Two mixing devices were tested: a vortex mixer and an ultrasonic probe. These have been previously used to produce porous materials from sol–gel^15,16^. Three solvents were employed in this study, viz. cyclohexane, hexane and 1-octanol (see “Optical microscopy evaluation of sol-gel mixture”(i) section). The other components of the mixture are described in “Optical microscopy evaluation of sol-gel mixture” section (and in footnote “e” of Table S1). The mixing time was 30 s for all experiments. It was found that mixing with the ultrasonic probe produced smaller uniform droplets compared with vortex mixing (see Fig. 2B(v)). Thus, the ultrasonic probe was chosen as the device for sol–gel mixing.

#### Final formulation and mixing method

From the results in “Optical microscopy evaluation of sol-gel mixture” section, the final selected formulation and procedure are given in  “Formation of silica layer coating on capillary wall by hydrothermal sol-gel process” section. This facile sol–gel process employs only one step synthesis for forming a layer coating on the inner wall surface of the silica capillary. It requires fewer steps for preparation of the coating than by the layer-by-layer procedure^10^.

### Characterization of the coating material

The sol–gel coating material was chemically characterized by Fourier-transform infrared spectroscopy (FT-IR) and X-ray powder diffraction (XRD). The surface morphology was studied by scanning electron microscopy (SEM) and contact angle (CA) measurements. The details of the instruments and preparation of the coating material are described in “Characterizations of sol-gel coating material” section.

#### Chemistry of coating layer material

**Figure 3:**
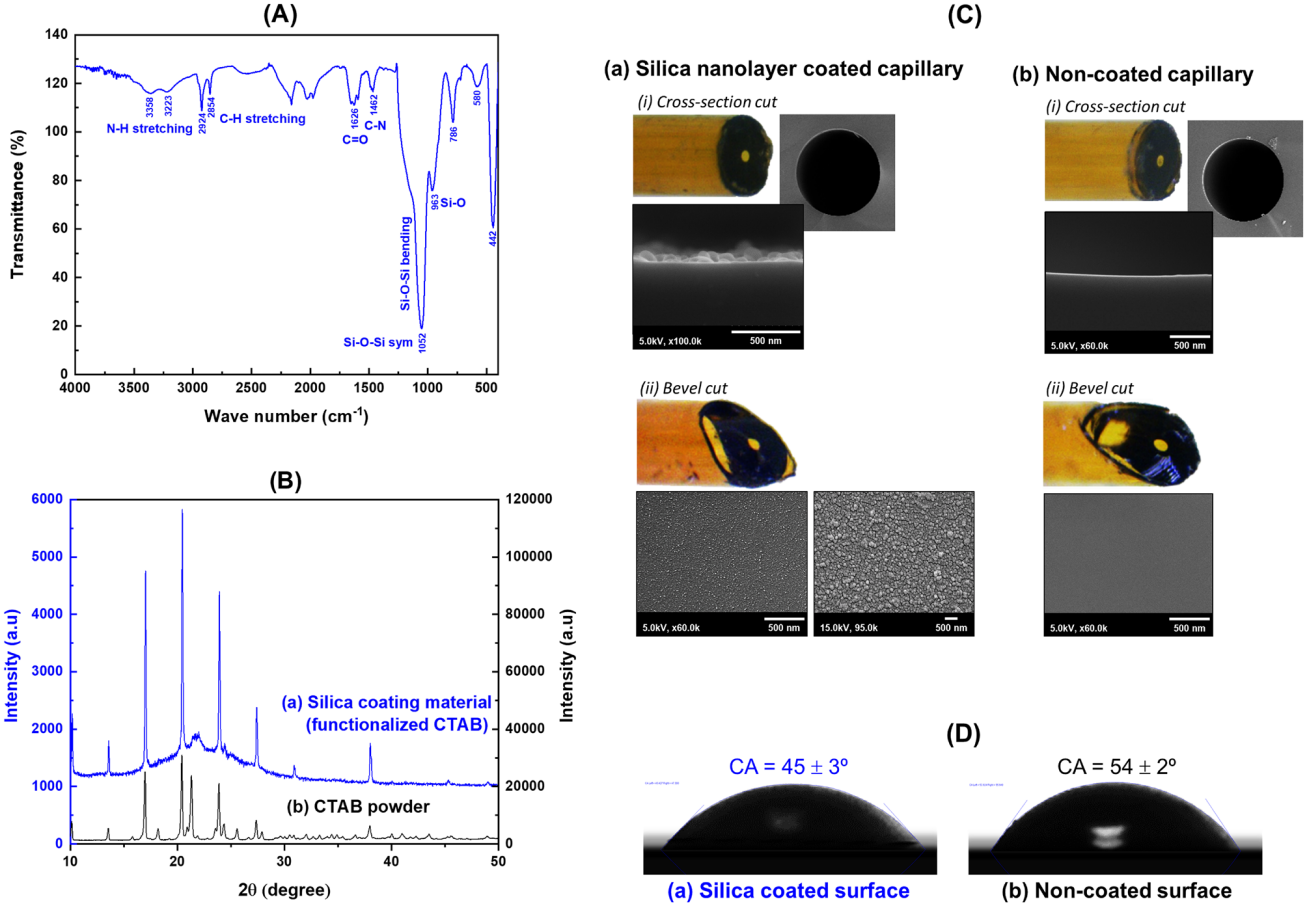
(**A**) FT-IR spectra of silica coated material (functionalized CTAB). The spectrum of the coating material is from a porous material prepared using the hydrothermal sol–gel procedure with the selected sol–gel formulation. (**B**) XRD patterns of (a) silica coating material (functionalized CTAB) and (b) CTAB powder. (**C**) *In-situ* SEM images of inner surface morphology of capillaries: (a) silica nanolayer coated capillary and (b) non-coated capillary. The thickness of the layer is observed from the horizontal cut (i) and the morphology of the surface observed from the bevel cut (ii). (**D**) Contact angle measurements of (a) silica coated surface (b) non-coated surface (glass slide used as substrate), showing the contact angles as measured by ImageJ software.

***FT-IR****:* IR absorbance bands of the coating material are observed at 786, 963 and 1052 cm^-1^, which are characteristics of the Si‒O, Si‒OH and Si‒O‒Si bonds, respectively (see Fig. 3A). In addition, peaks for urea at 1592 cm^-1^ (‒NH_2_) and CTAB at 1467, 2854 and 2923 cm^-1^ (C‒H bending and C‒H stretching) are also observed (see Supplementary Information C for FT-IR spectra of pure CTAB and urea).

***XRD***: Figure 3B(a) shows a broad low intensity XRD peak at diffraction angle *2θ* of 22.0°. This is the reflection of pure amorphous silica^37^. There are also sharp XRD peaks at angle *2θ* of 10.0, 13.6, 17.0, 20.4, 21.0, 23.8, 27.4 and 38.0°, respectively. These peaks are at the same positions as for powdered CTAB (see Fig. 3B(b). Thus, the XRD spectra of the coating material, together with the FT-IR data, show that CTAB is functionalized in the amorphous silica. Cationic CTAB surfactant has been reported to be adsorbed on the surface of silica nano particles synthesized by sol–gel process but the adsorption step was carried using the dispersed synthesized silica particles^38–40^. In this work CTAB is one of the components of the sol–gel formulation (see “Optical microscopy evaluation of sol-gel mixture”(iii) section) and thus it is not possible to state whether the CTAB is also on the surface. However, the results of the contact angle values (“Surface morphology” section) and EOF mobilities (“Properties of silica nanolayer coated capillary” section, non-coated and coated capillaries) confirm that the hydrophilicity of the surface of the silica coating has been modified as compared with the non-coated surface.

#### Surface morphology

Figure 3C(a)(i) shows SEM images of the cross-section cut of a capillary. A thin silica layer is observed deposited on the capillary inner wall with a thickness of *ca.* 100 nm. This is not present at the surface of non-treated capillary (see images in Fig. 3C(b)(i)). With the bevel cut, the SEM images in Fig. 3C(a)(ii) shows cobbled texture of the coating surface when using high resolution of 95,000 × magnification. The mean diameter of the dome-like protuberances is 23 ± 3 nm (*n* = 25), measured using ImageJ software. The coating surface also has roughness microstructure with material deposited uniformly (see SEM images in Fig. 3C (a)). The coating, with its roughness morphology and chemical compositions, affects the surface wettability^41^. As discussed previously, the material, functionalized with CTAB (see Fig. 3D(a)) increases the hydrophilicity of the coating material on surface which has a contact angle (CA) of 45 ± 3° (*n* = 6) (see Fig. 3D(a)), compared to the CA of the non-coated surface of 54 ± 2° (*n* = 6), respectively (see Fig. 3D(b)).

### Efficiency and resolution of amines separation by using coated- and non-coated capillaries

**Figure 4:**
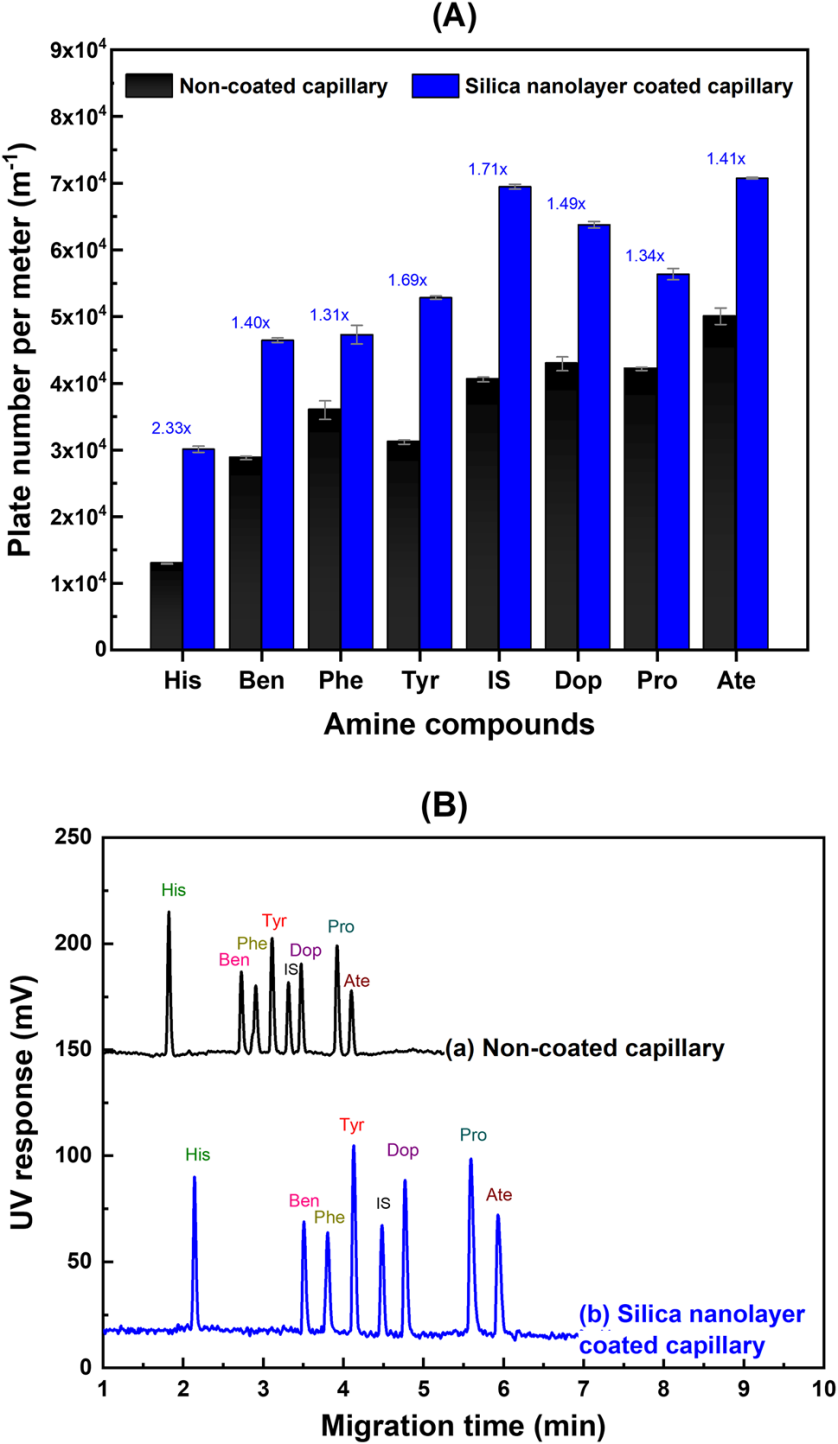
(**A**) Comparisons of plate number per meter for separation of eight amine compounds: histamine (His), benzylamine (Ben), phenylethylamine (Phe), tyramine (Tyr), benzhydrylamine (IS, internal standard), dopamine (Dop), propranolol (Pro) and atenolol (Ate). The numbers above the bar plots for silica nanolayer coated capillary are the fold-increase over the non-coated capillary. (**B**) Electropherograms of eight standard amines separation using (a) non-coated capillary and (b) silica nanolayer coated capillary. The concentrations are: His (400 μg L^-1^), Ben (200 μg L^-1^), Phe (100 μg L^-1^), Tyr (200 μg L^-1^), Dop (100 μg L^-1^), Pro (1000 μg L^-1^), Ate (500 μg L^-1^) and IS (100 μg L^-1^). The CEC conditions are: running buffer: 20.0 mM phosphate buffer (pH 2.5); electrokinetic injection: 400 V cm^-1^ for 3 s; field strength: 400 V cm^-1^; UV detection: 200 nm.

**Figure 5:**
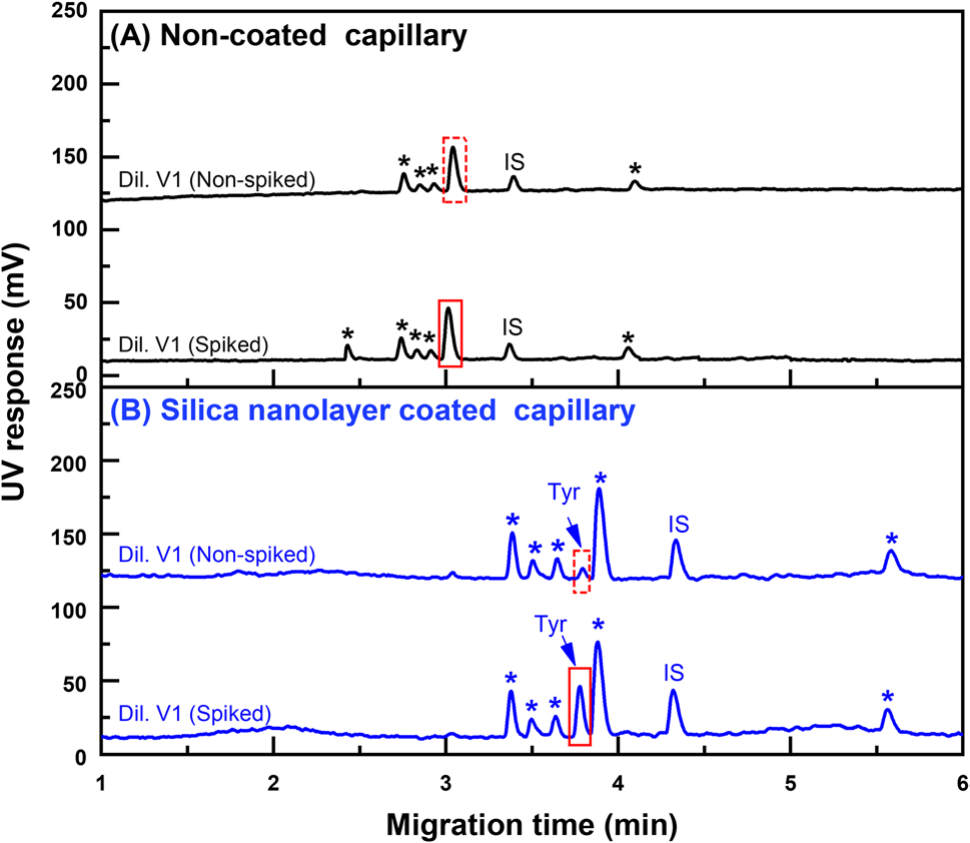
Electropherograms obtained from using two capillaries; (**A**) Non-coated capillary and (**B**) Coated silica nanolayer. The sample was analyzed using 20-fold diluted balsamic vinegar V1, sample spiked with standard tyramine at 50 μg L^-1^. The CEC conditions are: phosphate running buffer (20.0 mM, pH 2.5), 38.0 cm effective column length, 60.0 cm total column length, separation field strength of 400 V cm^-1^ (24 kV applied), electrokinetic injection for 3 s at 400 V cm^-1^, and absorbance measurement at 200 nm. Peak identification: **Tyr** (tyramine), **IS** (benzhydrylamine), * (unidentified peaks).

Separation efficiency expressed as plate number per effective capillary length (m^-1^) of silica nanolayer coated capillary for separation of eight amine compounds were calculated using the equation *N* = 5.54 × (t_m_ /W_1/2_)^2^ × (1/*L*_eff_), where t_m_ is the migration time (s), *L*_eff_ is the effective length of the capillary and W_1/2_ is the peak width at half maximum height. Figure 4A shows bar graphs of the plate number *N* between coated and non-coated capillaries for all 8 amines. Figure 4B shows the electropherograms using (a) non-coated and (b) coated capillaries, respectively (see “Capillary electrophoresis with UV detection” section for the CEC conditions). The coated capillaries have plate numbers *N* ≥ 3.0 × 10^4^ m^-1^ and resolutions *R*_s_ of all consecutive pairs of peaks of 2.69–13.94 (see Fig. 4B(b)), whereas the non-coated capillary has *N* ≥ 1.3 × 10^4^ m^-1^ with *R*_s_ of 1.49–9.23 (see Fig. 4B(a)). The increment of 1.3 to 2.3-fold of *N* gives narrower peaks, increased peak heights and 1.5 to 1.8-fold increase in *R*_s_. Thus, the coated capillary will have greater separation performance when applied to analysis of real samples (see Fig. 5 for example).

### Properties of silica nanolayer coated capillary

***Reproducibility of coating layer for capillary and its uniform coating:*** Reproducibility of the layer coating process was evaluated using EOF mobility measurements. Three coated capillaries were selected from different batches of coating procedures. The EOF mobility was measured by using phosphate buffer (20.0 mM, pH 2.5) and field strength of 400 V cm^-1^ (details of the EOF measurement is given in Supplementary Information D). To determine the uniformity of the coating along the length of the capillary, one coated capillary 60 cm long was divided into 3 segments and a 10.0 cm section from each segment used for EOF measurements. The mobility and SD (*n* = 5) of the three 10-cm sections are (0.62 ± 0.02), (0.61 ± 0.02) and (0.63 ± 0.02) × 10^–4^ cm^2^ V^-1^ s^-1^, respectively (see Table 3). The mean of the EOF of the three segments (left, middle and right) is (0.62 ± 0.01) × 10^–4^ cm^2^ V^-1^ s^-1^. The values of the mobilities of the three sections lie within the range of mean ± 3SD, indicating that the coating is uniform throughout the length of the 60 cm capillary. Similarly, the mobility and SD (*n* = 5*)* of three selected individual coated capillaries are (0.60 ± 0.01), (0.59 ± 0.01) and (0.56 ± 0.02) × 10^–4^ cm^2^ V^-1^ s^-1^, respectively (see Table 3).

**Table 3:** EOF mobilities of silica nanolayer coated capillaries: (1) a single coated capillary cut into three segments (left, middle, right); (2) one capillary selected from three batches.

Silica nanolayer coated capillary	EOF ± SD, × 10^–4^ cm^2^ V^-1^ s^-1^ (*n* = 5)
**(1) Capillary cut from 3 segments of a 60 cm capillary**	
Left segment (10.0 cm) Middle segment (10.0 cm) Right segment (10.0 cm)	0.62 ± 0.02 0.61 ± 0.02 0.63 ± 0.02
Mean	0.62 ± 0.01
**(2) One capillary (60 cm) from three batches**	
Capillary from batch #1 Capillary from batch #2 Capillary from batch #3	0.60 ± 0.01 0.59 ± 0.01 0.56 ± 0.02
Mean	0.58 ± 0.02

Measured EOF mobility of non-coated capillary (60.0 cm length) is 1.26 ± 0.01 cm^2^ V^-1^ s^-1^ (*n* = 5).

The mean EOF mobility from the three capillaries is (0.58 ± 0.02) × 10^–4^ cm^2^ V^-1^ s^-1^. Thus, the values of the mobilities are within ± 3SD of this mean value, confirming no statistical differences between the EOF mobility of the capillaries. The mobility of a 60 cm non-coated capillary is 1.26 ± 0.01 cm^2^ V^-1^ s^-1^ (see Table 3).

***Precision of migration time***: Precision of the relative migration time (RMT) of 50 μg L^-1^ standard tyramine, with 100 μg L^-1^ benzhydrylamine as internal standard, was determined (see “Capillary electrophoresis with UV detection” section) using three 60.0 cm coated capillaries. The mean and standard deviation (SD) of RMTs from 10 repeated injections are 0.86 ± 0.01, 0.86 ± 0.02 and 0.86 ± 0.01 for the three capillaries, respectively. Paired *t*-tests for all pairs of capillaries show no statistical difference between each pair: *t*_*stat*_ = 0.39, for capillary#1 and capillary#2; *t*_*stat*_ = 0.58, for capillary#1 and capillary#3; *t*_*stat*_ = 1.14, for capillary#2 and capillary#3; *t*_*crital*_ = 1.72, for *α* = 0.05.

***Stability of coated capillary***: To investigate the stability of the coating, a solution of 200 μg L^-1^ standard tyramine with 100 μg L^-1^ benzhydrylamine (IS) was injected consecutively 35 times over a period of *ca.* 10 h. This was repeated on two further days. The %RSD of the mean of the relative migration times of a set of 35 consecutive injections per day was 1.3%, with the absolute migration times decreasing by ca. 16% from the first to the 105th injection. Thus, the capillary can be used for at least 105 separations.

### Detection of tyramine in food products using silica nanolayer coated capillary

#### Analytical characteristics: calibration, limit of detection and precision

The linear calibration range of tyramine was 5.00–200 μg L^-1^, with calibration equation of peak area ratio with concentration of *y* = (0.0183 ± 0.0003)*x* + (0.11 ± 0.03), and coefficient of determination (r^2^) of 0.9985. The instrumental LOD calculated from 3 × (SD of intercept)/(slope of calibration line) was 4.9 μg L^-1^^42^.

Intra-day and inter-day precisions of quantitation of tyramine using the coated capillary was determined from triplicate injections of standard tyramine solutions of 20, 200 and 400 μg L^-1^, respectively, on three different days using the same capillary and operating conditions. The intra-day and the inter-day precisions are 0.03–0.41%RSD and 0.75–1.22%RSD, respectively. The relative migration times from three replicate injections of five samples are less than 1.8%RSD (see Table 4).

**Table 4:** Tyramine concentration of diluted samples, recoveries of spiked diluted samples, relative migration times (RMT) and content in samples.

Sample	Tyramine concentration (μg L^-1^)			Percent recovery ± SD	RMT ± SD, *n* = 3 (%RSD)	Tyramine in sample ± SD, *n* = 3 (μg L^-1^)
	Diluted sample ± SD, *n* = 3 (Dilution factor)	Spiked concentration	Spiked diluted sample ± SD, *n* = 3			
B1	ND^a^ (426)	50	52.3 ± 1.6	105 ± 3	0.87 ± 0.002 (0.20%)	ND^b^
B2	7.7 ± 0.5 (638)	50	48.4 ± 5	97 ± 10	0.86 ± 0.001 (0.15%)	4900 ± 300
W1	ND^a^ (1)	50	47.4 ± 1.6	95 ± 3	0.87 ± 0.001 (0.12%)	ND^b^
V1	53.6 ± 2.5 (20)	50	106 ± 4	106 ± 7	0.87 ± 0.002 (0.22%)	1080 ± 50
C1	37.4 ± 1.9 (40)	30	67 ± 3	97 ± 9	0.83 ± 0.014 (1.72%)	1500 ± 76^c^

Dilution factors for all samples were 426 × , 638 × , 1 × , 20 × and 40 × for B1, B2, W1, V1 and C1, respectively.

^a^ND; Not Detected (< LOD of 4.9 μg L^-1^).

^b^ND; Not Detected (< sample LOD of 2.1 μg L^-1^).

^c^Extracted amount from hard cheese.

#### Detection of tyramine in food products, recovery study and method comparison

Four different fermented products were analyzed, viz. beer, wine, balsamic vinegar and cheese (see “Food and drink products” section). All liquid samples were diluted with ultrapure water using aliquots of 1.0–30.0 µL (Repeater® E3 pipette, Eppendorf, Hamburg, Germany) of sample with a final volume of 600.0 µL. The cheese sample was extracted using the procedure described in Supplementary Information E.

The samples are two brands of beer (B1, B2), wine (W1), balsamic vinegar (V1) and hard cheese (C1). Various dilution factors were used as given in the footnote of Table 4. Figure 5 shows examples of electropherograms of sample V1 after 20-fold dilution using the non-coated and silica nanolayer coated capillaries. The electropherograms are the diluted balsamic vinegar sample V1 and the same sample spiked with standard tyramine at 50 μg L^-1^ (both with 100 μg L^-1^ IS). It can be clearly seen that the tyramine peak is not resolved from matrix peaks using non-coated capillary (see Fig. 5A), while the coated capillary is able to resolve the tyramine peak from the matrix peaks (see Fig. 5B). As observed in Fig. 5A there is only a small change in the peak in the region of the migration time of tyramine after spiking. (see peak inside box with the solid line compared with peak inside the box with the dotted line). In contrast, the identity of the tyramine peak in the electropherogram of Fig. 5B is confirmed by the increase in peak height after spiking (see peak in box with solid line compared with peak inside box with dotted line). Table 4 lists the measured tyramine concentrations of the diluted samples, the recoveries of spiked tyramine standard, the relative migration times and the calculated tyramine contents in the samples. The percent recovery of the spiked diluted sample was calculated from % recovery = [[(*S*_1_ − *S*_2_)/*S*_0_] × 100], where *S*_0_ is the peak area ratio of the standard tyramine solution, *S*_1_ is the peak area ratio of spiked diluted sample, and *S*_2_ is the peak area ratio of the diluted sample. Percent recoveries are in the range of 95 ± 3–106 ± 7% (*n* = 3).

Table 2 lists the comparison of previous reports using CE-UV methods for tyramine determination with this work. The performance of the present work employing coated silica nanolayer capillary shows wider dynamic range and a lower LOD value for comparisons with some works. Different sample pre-treatments, such as liquid- or solid-extraction and dilution, were required for preparation of the samples. This work has faster separation than the other works, high separation efficiency and precision, and reasonable percent recovery. Thus, it is suitable for detection of low levels of tyramine, especially of food for patients taking antidepressant drugs.

## Conclusion

An optimized formulation for a sol–gel mixture that provides reproducible and uniform nanolayer silica coating on the wall of a capillary by a hydrothermal process was successfully developed. Recorded images of the sol formation from an optical microscope was employed to select the optimal formulation based on the observed size distribution of the immiscible droplets, together with the gelling time of the mixture. The selected formulation and mixing procedure produced coating of *ca.* 100 nm thickness layer on the inner wall of capillaries. The coating was characterized by in-situ SEM, contact angle, FT-IR spectroscopy and X-ray powder diffraction. The SEM images revealed cobbled texture of the coated surface. The FT-IR spectra and X-ray powder diffraction pattern indicated that CTAB was incorporated in the layer material. The contact angle data showed that CTAB on the coating increased the surface hydrophilicity.

EOF measurements were carried out to evaluate the reproducibility and stability of the coated capillaries. Comparison of capillary performance for separation of amines between nanolayer coated and conventional non-coated capillaries are presented with regards to efficiency, resolution, as well as EOF mobilities. The nanolayer coated capillary provides higher efficiency and consequently higher peak heights for the separation of eight standard amines, with plate number *N* of ≥ 3.0 × 10^4^ m^-1^ and higher peak resolution, *R*_s_ ≥ 2.69 for all adjacent pairs of peaks. Using the coated capillary, precision of the relative migration time (RMT) for tyramine (with benzhydrylamine as IS) is < 1.3%RSD for 35 injections. Inter-day precision over 3 days was < 1.2%RSD. The nanolayer coated capillary was applied to the analysis of tyramine in food and drinks and the comparison of our method with other works is listed (Table 2). The method provides lower LOD than previous works, wide dynamic range and high precision (%RSD of RMT < 2%) and high separation efficiency for tyramine. Accuracy based on percent recoveries of spiked diluted samples were in the range of 95 ± 3–106 ± 7%.

## Supplementary Information

Supplementary Information.
