# Supplementary material for: Silica nanolayer coated capillary by hydrothermal sol–gel process for amines separation and detection of tyramine in food products
Source: Sci Rep. 2022 May 6;12:7460. doi: 10.1038/s41598-022-11078-y (PMC9076594; doi:10.1038/s41598-022-11078-y)
Supplement: Supplementary file 1 — Supplementary Information. [file 41598_2022_11078_MOESM1_ESM.docx]

**Supplementary Information**

**Silica Nanolayer Coated Capillary by Hydrothermal Sol-gel Process for Amines Separation and Detection of Tyramine in Food Products**

Apinya Obma^1,2^, Pattamaporn Hemwech^1,2^, Sittisak Phoolpho^1,2^, Rawiwan Bumrungpuech^1,2^,

Supa Wirasate^1,3^, Sulawan Kaowphong^4^, Prapin Wilairat^5^, Rattikan Chantiwas^1,2*^

^1^Department of Chemistry, Faculty of Science, Mahidol University, Rama VI Rd., Bangkok 10400, Thailand

^2^Center of Excellence for Innovation in Chemistry and Flow Innovation-Research for Science and Technology Laboratories (FIRST Labs), Faculty of Science, Mahidol University, Rama VI Rd., Bangkok 10400, Thailand

^3^Center for Surface Science and Engineering and Rubber Technology Research Center, Faculty of Science, Mahidol University, Salaya, Nakhorn Pathom 73170, Thailand

^4^Department of Chemistry and Environmental Science Research Center (ESRC), Faculty of Science, Chiang Mai University, Chiang Mai 50200, Thailand

^5^Analytical Sciences and National Doping Test Institute, Mahidol University, Rama VI Rd., Bangkok 10400, Thailand

*Corresponding author. Tel.: +66 2 201 5199; Fax: +66 2 354 7151

E-mail: rattikan.cha@mahidol.ac.th, rattikan.cha@mahidol.edu

**Contents:**

1. **Supplementary Information A:** Investigation of composition and mixing method for hydrothermal sol-gel production
2. **Supplementary Information B:** Procedures for preparation of sol-gel coating material, capillary cutting for SEM and contact angle measurement
3. **Supplementary Information C:** FT-IR measurements of pure urea and cetyltrimethyl-ammonium bromide (CTAB)
4. **Supplementary Information D:** Measurement of EOF mobility of silica nanolayer coated and non-coated capillaries by contactless conductivity detection
5. **Supplementary Information E:** Sample preparation of hard cheese

**Supplementary Information A**

**Investigation of composition and mixing method for
hydrothermal sol-gel production**

**Table S1** List of components and final composition of the sol-gel formulation.

| **Component** | **Selection** | **Final composition** |
| --- | --- | --- |
| 1. Solvent *^a^* | Hexane, Pentane, Toluene, Cyclohexane, 1-Octanol, Benzene, TBME, DMSO | Cyclohexane |
| 1. Water-TEOS mole ratio *^b^* | 4.0, 6.0, 8.0 | 6.0 |
| 1. CTAB content (mg) *^c^* | 0, 50, 100, 150, 200 | 100 |
| 1. Urea content (mg) *^d^* | 20, 40, 60, 80, 100 | 60 |
| 1. Mixing method *^c^*^,^ *^e^* | Vortex mixer  Ultrasonicator | Ultrasonicator |

*^a^* Sol-gel formulation: TEOS: 500 µL (0.0259 mol), water-TEOS mole ratio: 6.0, CTAB: 100 mg, urea: 60 mg, 0.10 mM acetic acid: 440 µL, 1-pentanol: 92 µL, vortex (30 s). Volume of solvent: 3 mL., *^b^* Sol-gel formulation: cyclohexane: 3 mL, CTAB: 100 mg, urea: 60 mg, 0.10 mM acetic acid: 440 µL, 1-pentanol: 92 µL, ultrasonication (20 Watt power, 30 s). Water-TEOS mole ratio: 4.0, 6.0 and 8.0 TEOS: 500 µL (0.0259 mol), water: 2 mL (0.1111 mol), 3 mL (0.1667 mol) and 4 mL (0.2222 mol)., *^c^* Sol-gel formulation: cyclohexane: 3 mL, TEOS: 500 µL (0.0259 mol), water: 3 mL (0.1667 mol), water-TEOS molar ratio: 6.0, urea content: 60 mg, 0.10 mM acetic acid: 440 µL, 1-pentanol: 92 µL, ultrasonication (20 Watt power, 30 s)., *^d^* Sol-gel formulation: cyclohexane: 3 mL, TEOS: 500 µL (0.0259 mol), water: 3 mL (0.1667 mol), water-TEOS molar ratio: 6.0, CTAB: 100 mg, 0.10 mM acetic acid: 440 µL, 1-pentanol: 92 µL, ultrasonication (20 Watt power, 30 s)., *^e^* Sol-gel formulation: 1-octanol, cyclohexane or hexane: 3 mL, TEOS: 500 µL (0.0259 mol), water: 3 mL (0.1667 mol), water-TEOS molar ratio: 6.0, CTAB: 100 mg, urea content: 60 mg, 0.10 mM acetic acid: 440 µL and 1-pentanol: 92 µL, mixing time: 30 s. **Note:** Density of TEOS = 0.933 g mL^-1^.

**Table S2** Physical properties of the organic solvents

| **Solvent** | **Solubility in water**  **(g/100 g)** | **Boiling point (°C)** | **Polarity index** |
| --- | --- | --- | --- |
| Hexane | 0.0014 | 59.0 | 0.1 |
| Pentane | 0.004 | 36.1 | 0.0 |
| Toluene | 0.05 | 110.6 | 2.4 |
| Cyclohexane | 0.0055 | 80.7 | 0.2 |
| 1-Octanol | 0.096 | 195.0 | 3.4 |
| Benzene | 0.18 | 80.1 | 2.7 |
| TBME | 4.80 | 55.2 | 2.4 |
| DMSO | *miscible* | 189.0 | 7.2 |


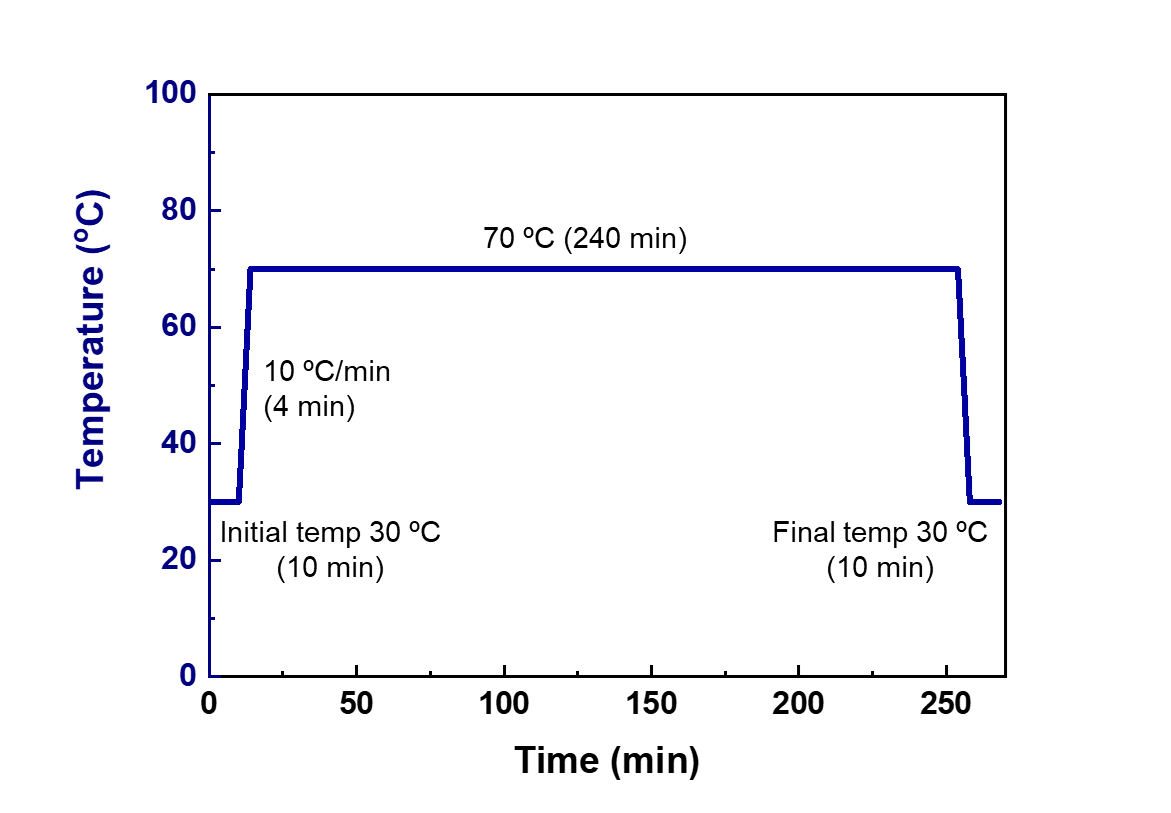


**Figure S1** Oven temperature program for hydrothermal sol-gel coating of capillary. Initial temperature, 30 ºC, hold 10 min, increase to 70 ºC at 10 ºC min^-1^, hold 240 min, decrease to 30 ºC, hold 10 min.

**Supplementary Information B**

**Procedures for preparation of sol-gel coating material, capillary cutting for SEM and contact angle measurement**

**Preparation of sol-gel coating:** The sol-gel mixture (see Section “Formation of silica layer coating on capillary wall by hydrothermal sol–gel process”) was heated at 70 °C for 4 h to form a powder. The resulting material was cooled to room temperature. The powder was then characterized by FT-IR and XRD (see Section “Chemistry of coating layer material” for the results of the chemical characterization).

**SEM:** Cutting capillary is performed for SEM characteristic; *(i)* Cutting of capillary: The two methods of capillary cutting are (a) cross-section (horizontal) cut using diamond blade cutter (Shortix, Sigma-Aldrich Co., USA) and (b) bevel cut using ceramic capillary cutter (Agilent Technologies Inc., DE, USA). Figure S2 shows the shape of the cut.

**
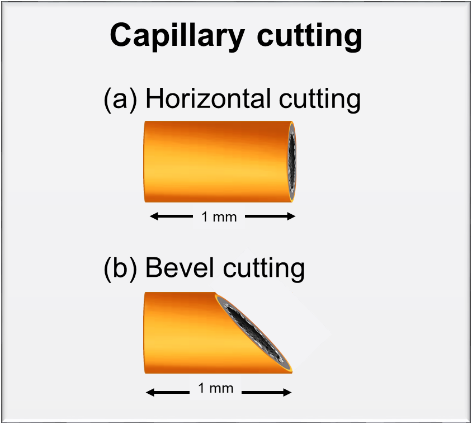

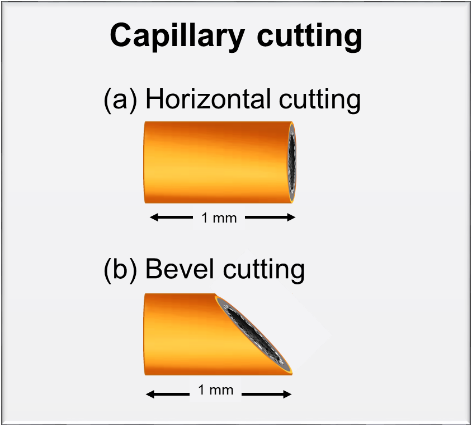
**

**Figure S2** Schematics of capillary cuts: (a) cross-section (horizontal) and (b) bevel cutting. The bevel cut is employed for characterization of *in-situ* surface morphology.

**Contact angle (CA):** Glass slides were first cleaned by immersing sequentially in MeOH-H_2_O (50 % v/v) (5 min); 1.0 M NaOH (30 min) and ultrapure water (5 min). The clean glass slide was then dip-coated with the sol-gel mixture (see Section “Formation of silica layer coating on capillary wall by hydrothermal sol–gel process”). The coated slide was dried in an oven at 70 °C for 4 h, cooled to room temperature and rinsed with EtOH and ultrapure water.

The left and right contact angles of the water drops were measured using ImageJ software with Plugin: Drop analysis – DropSnake (see Section “Surface morphology”, Fig. 3D).

The operation steps are as follows:

Step 1. Open digital image file: go to File 🡪 Open from menu bar to open a digital image, as shown in picture below.


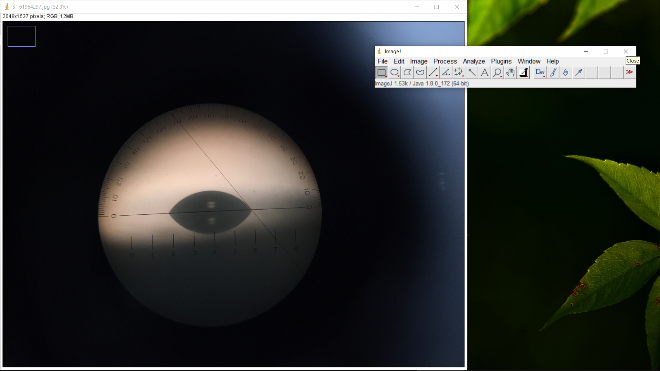


Step 2 Convert digital color image (RGB) into gray scale: scale “Image 🡪 Type 🡪 32-bit”, as shown in picture below.


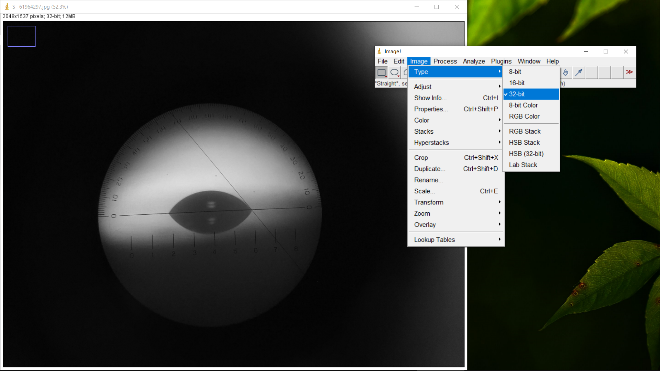


Step 3. Measure the contact angle (CA) of the water drop using Plugin: Drop analysis-DropSnake: Go to “Plugins 🡪 Drop analysis – DropSnake”. Adjust the knots positions (8 - 10 knots) to perfectly fit with the curvature of the image of the drop. The software reports the CA at display at the top left of the image, as shown in picture below.


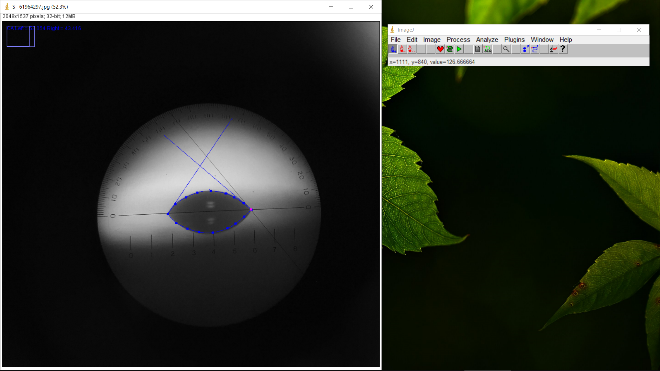


**Supplementary Information C**

**FT-IR measurements of pure urea and cetyltrimethylammonium bromide (CTAB)**

FT-IR spectra of pure urea and CTAB were recorded using attenuated total reflection (ATR) accessory on FT-IR spectrometer (INVENIO®, Bruker) in the range of 4000–400 cm^-1^. Before the measurement, urea and CTAB were dried in an oven at 50 °C for 3 h.

Characteristic bands of CTAB and urea are shown in Fig. S3. The bands for urea are at 1456 cm^-1^ and 1466 cm^-1^ (C‒N stretching), 1592 cm^-1^ (N‒H deformation), 1678 cm^-1^ (C=O stretching) and 3428 cm^-1^ (N‒H stretching). The bands for CTAB are at 1467 cm^-1^ and 1487 cm^-1^ (C‒N stretching), 1473 cm^-1^ (methylene group), 2854 cm^-1^, 2923 cm^-1^ (C‒H stretching) and 3018 cm^-1^ (N‒H stretching).

**Figure S3** FT-IR spectra of pure urea and CTAB.

**Supplementary Information D**

**Measurement of EOF mobility of silica nanolayer coated and non-coated capillaries by contactless conductivity detection**

Measurements of EOF mobility of silica nanolayer coated and conventional non-coated and capillaries by contactless conductivity detection were carried out. Details of instruments, conditions and measurement are given below.

**Capillary electrophoresis with contactless conductivity detection**

The CE system was assembled in-house and the capillary connected to a contactless conductivity detector (C^4^D, eDAQ ET 120, Denistone East, NSW, Australia). The CE system is placed in a Plexiglass box with a micro switch connected to the ON/OFF control of the high voltage power supply unit (Spellman CZE1000R, Hauppauge, USA). The signal (mV) from the C^4^D detector is recorded by a data acquisition system (eDAQ, Denistone East, NSW, Australia) with eDAQ Chart software. Non-coated and coated capillaries were cut into 10.0 cm lengths and placed into the CE system. Effective length of the capillary was 5.0 cm. Before use, the capillaries were conditioned by sequentially flushing with 0.1 M NaOH, water and running buffer (5 min each), using a manual syringe pump (Unimicro Technologies, CA, USA). The applied voltage for EOF measurement was set to give a field strength of 200 - 450 V cm^-1^ (See Section “Efficiency and resolution of amines separation by using coated- and non-coated capillaries” and “Properties of silica nanolayer coated capillary”).

After conditioning, the capillary is filled with a buffer with a relatively lower conductivity (20.0 mM phosphate buffer at pH 2.5). The conductivity of the buffer in the capillary is monitored by the contactless conductivity detector at a certain distance from the inlet end of the capillary. The inlet container is filled with the same buffer but at the concentration of 40.0 mM. A potential is applied and the time at which there is a sharp increase in conductivity (the EOF migration time) is noted (see Fig. S4). The EOF mobility is calculated from (*LL*_eff_ / *Vt*), where *L* (cm) is the total length of the capillary, *L*_eff_ (cm) is the distance between the capillary inlet and detector, *V* is the voltage applied across the capillary and *t* is the migration time (s) of the EOF. The unit of mobility is cm^2^ V^-1^ s^-1^.

**Figure S4** Schematic representation of the conductivity signal with time in the measurement of EOF migration time by contactless conductivity measurement showing the time at conductivity increase.

**Supplementary Information E**

**Sample preparation of hard cheese**

The hard cheese sample was extracted as follows: hard cheese (10.0 g) was placed in a centrifuge tube containing 20.0 mL of 0.10 M HCl, mixed on a vortex mixer (5 min) and centrifuged (Rotofix 32A, Hettich Zentrifugen, Germany) at 5000 rpm for 30 min at room temperature. The supernatant was collected. The extraction procedure was repeated 3 times with addition of 20.0 mL of 0.10 M HCl. All the supernatants (60.0 mL) were combined and cooled to 4 °C to allow the solidification of fats, which were then filtered out through a Whatman no. 1 filter paper at 25 ^o^C. The supernatant was then diluted 40-fold using ultrapure water (final volume of 10.0 mL), with an addition of benzhydrylamine at concentration 100 μg L^-1^ as the internal standard, before introducing into the CE-UV system.
